# SARS-CoV-2 Next Generation Sequencing (NGS) data from clinical isolates from the East Texas Region of the United States

**DOI:** 10.1016/j.dib.2023.109312

**Published:** 2023-06-14

**Authors:** Rob E. Carpenter, Vaibhav K. Tamrakar, Sadia Almas, Aditya Sharma, Rahul Sharma

**Affiliations:** a Advanta Genetics, 10935 CR 159, Tyler, TX 75703, USA; b University of Texas at Tyler, 3900 University Boulevard, Tyler, TX 75799, USA; c ICMR-National Institute of Research in Tribal Health, Jabalpur, MP 482003, India; d RetroBioTech LLC, 838 Dalmalley Ln, Coppell, TX 75019, USA

**Keywords:** SARS-CoV-2, qRT-PCR, Next generation sequencing, Variants of concern, Epidemiology, Transmission dynamics

## Abstract

The SARS-CoV-2 virus has evolved throughout the pandemic and is likely to continue evolving into new variants. Some of these variants may affect functional properties, including infectivity, interactions with host immunity, and disease severity. And compromised vaccine efficacy is an emerging concern with every new viral variant. Next-generation sequencing (NGS) has emerged as the tool of choice for discovering new variants and understanding the transmission dynamics of SARS-CoV-2. Deciphering the SARS-CoV-2 genome has enabled epidemiological survivance and forecast of altered etiologically. Clinical presentations of the infection are influenced by comorbidities such as age, immune status, diabetes, and the infecting variant. Thus, clinical management and vaccine efficacy may differ for new variants. For example, some monoclonal antibody treatments are variant-specific, and some vaccines are less efficacious against the omicron and delta variants of SARS-CoV-2. Consequently, determining the local outbreaks and monitoring SARS-CoV-2 Variants of Concern (VOC) is one of the primary strategies for the pandemic's containment. Although next-generation sequencing (NGS) is a gold standard for genomic surveillance and variant discovery, the assays are not approved for variant diagnosis for clinical decision-making. Advanta Genetics, Texas, USA, optimized Illumina COVID-seq protocol to reduce cost without compromising accuracy and validated the Illumina COVID-Seq assay as a Laboratory Developed Test (LDT) according to the guidelines prescribed by the College of American Pathologists (CAP) and Clinical Laboratory Improvement Amendments (CLIA). The whole genome of the virus was sequenced in (*n* = 161) samples from the East Texas region using the Illumina MiniSeq® instrument and analyzed by using Illumina baseSpace (https://basespace.illumina.com) bioinformatics pipeline. Briefly, the library was prepared by using Illumina COVIDSeq research use only (RUO) kit, and the individual libraries were normalized using the DNA concentration measured by Qubit Flex Fluorometer, and the pooled libraries were sequenced on Illumina MiniSeq® Instrument. Illumina baseSpace application was used for sequencing QC, FASTQ generation, genome assembly, and identification of SARS-CoV-2 variants. This whole genome shotgun project (*n* = 161) has been deposited at GISAID.

**Specifications Table**

**Table:** 

Subject	Health and Medical sciences
Specific subject area	Genomic: Virology
Type of data	Raw FASTQ files; available at GISAID; Figure; Table (supplementary)
How the data were acquired	This data was acquired by sequencing of SARS-CoV-2 samples from the PCR-positive patient samples from the East Texas region. Sequencing data were analyzed by using DRAGEN COVID Lineage Basespace Labs (3.5.4). Analyzed data has been published [1,2].
Data format	Raw data (fastq files accession ID), Analyzed data for surveillance over the time; Filtered by coverage
Description of data collection	Nasopharyngeal swab samples were collected from suspected SARS-CoV-2 cases and tested for SARS-CoV-2 by qRT-PCR. Only positive (∼30 Ct values) samples were selected for SARS-CoV-2 whole genome sequencing using the COVIDSeq protocol and the MiniSeq® (Illumina) instrument. The term "Ct" in the context of a real-time reverse transcription PCR (qRT-PCR) assay refers to the cycle threshold, which is the number of times a machine attempts to replicate the genetic material of a given virus before it is successfully detected in the given sample. This value indirectly measures the amount of viral genetic material present in the specimen at the time of testing. A lower Ct value indicates a higher viral load, while a higher Ct value indicates a lower viral load. In general, samples with Ct values below 32 are considered suitable for whole genome sequencing (WGS) and are more likely to contain replicable virus.
Data source location	Institution: Advanta Genetics City/Town/Region: Smith County, East Texas region Country: United States Latitude and longitude: 31.9686° N, 99.9018° W
Data accessibility	Data Availability GISAID Identifier: EPI_SET_20220715vh All genome sequences and associated metadata in this dataset are published in GISAID's EpiCoV database. To view the contributors of each sequence with details such as accession number, virus name, collection date, originating lab and submitting lab, and the list of authors, visit https://doi.org/10.55876/gis8.220715vh
	Data Snapshot EPI_SET_20220715vh is composed of 72 individual genome sequences. The collection dates range from 2020-08-01 to 2022-09-25; Data were collected in 1 country and territory; All sequences in this dataset are compared to hCoV-19/Wuhan/WIV04/2019 (WIV04), the official reference sequence employed by GISAID (EPI_ISL_402124). Learn more at https://gisaid.org/WIV04. Repository name: GISAID: Global Initiative on Sharing All Influenza Data
Related research article(s)	Rob E. Carpenter, Vaibhav Tamrakar, Harendra Chahar, Tyler Vine, Rahul Sharma (2022). Confirming Multiplex RT-qPCR Use in COVID-19 with Next-Generation Sequencing: Strategies for Epidemiological Advantage, *Global Health, Epidemiology and Genomics*, vol. 2022, Article ID 2270965, 1-10. https://doi.org/10.1155/2022/2270965

## Value of the Data

• This data may be useful to researchers mapping the evolution of SARS-CoV-2 variant mutations from the East Texas region, as well as the efficacy of diagnostic techniques for variant calling.
• The data can be useful for researchers working on circulating variants of SARS-CoV-2. This study may help to develop strategies and control programs for this pandemic.
• The data will be used to retrieve information about the circulating and dominating strains of SARS-CoV-2, which may help better understand the transmission dynamics of SARS-CoV-2 and develop strategies for preventing the pandemic from returning. Moreover, the data can be used to develop genomics-derived transmission prediction models to predict infectious disease spread in the future. Several studies have suggested the differential clinical presentation and vaccine efficacy for different SARS-CoV-2 strains, which may guide therapeutic decisions, such as monoclonal antibody therapies and future vaccination strategies [3,4].

## 1 Objective

Primary objective for this data acquisition was to monitor the evolution of SARS-CoV-2 variants in the East Texas regions. Second objective was to establish the SARS-CoV-2 variant detection as Laboratory Developed Test (LDT) for clinical reporting.

## 2 Data Description

**Fig. 1:**
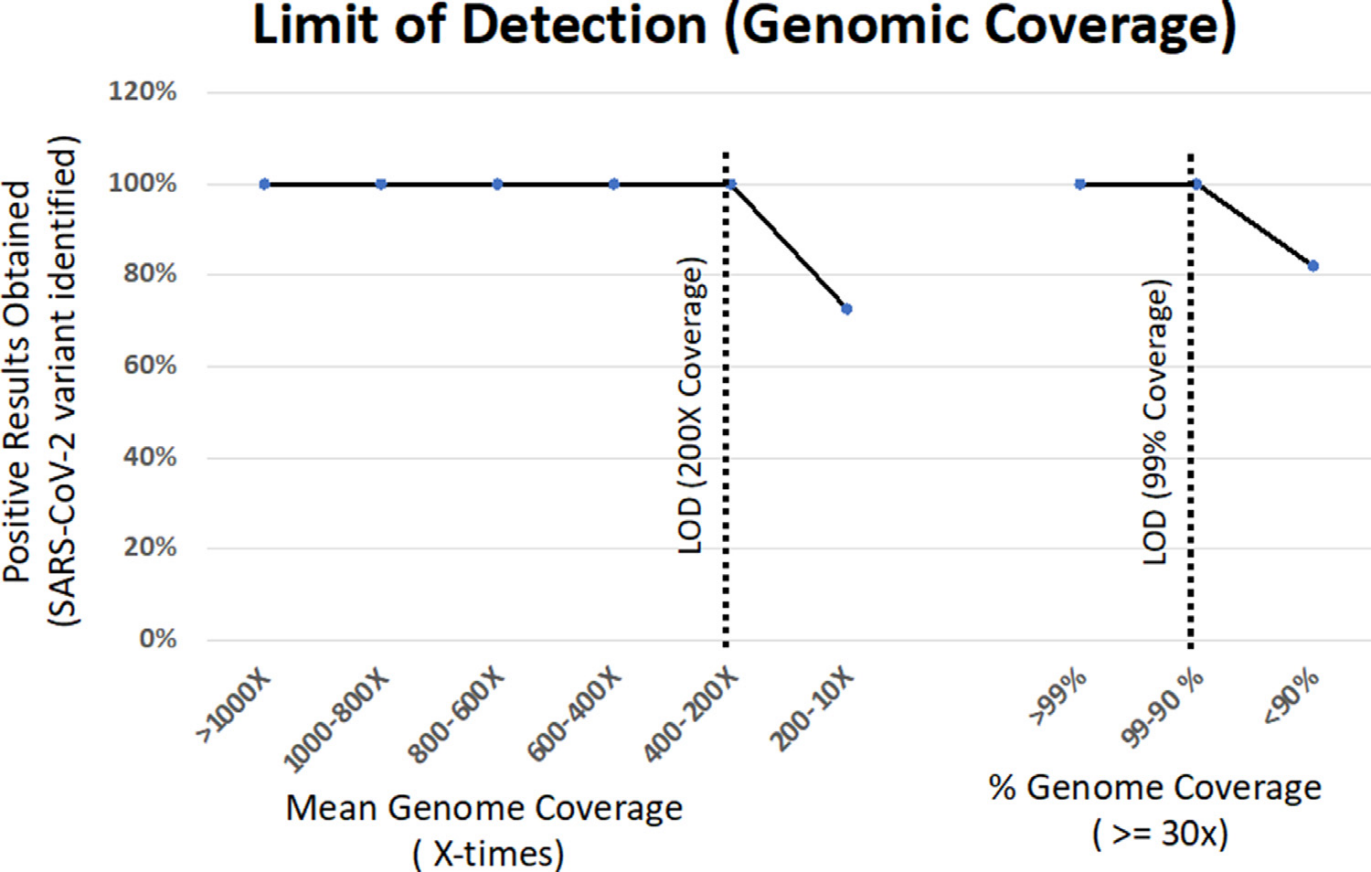
Mean genomic coverage (X-times) and minimum % length of the genome covered >30X times were computed, and the minimum coverage required for obtaining the accurate SARS CoV-02 lineage was defined as LOD. *Reused from published article and associated raw data is available as supplementary table [1,sheet-1]*.

Here we present whole genome sequencing data obtained from 161 qRT-PCR positive SARS-CoV-2 nasopharyngeal samples from the East Texas region collected between August 2020 and September 2022. Data repository: **GISAID: EPI_SET_20220715vh** [1]. Data was analyzed after determining the limit of detection (LOD) of genomic coverage by computing the depth of coverage (X times) and percent genome coverage for all tested samples. The lowest genomic coverage of >200X (depth) and 90% genome coverage was required for successful detection variant detection (Fig. 1). Importantly, all 161/161 (100%) observations with a minimum of 90% genome coverage at a minimum of 200X resulted in the correct variant call after the analysis.

**Fig. 2:**
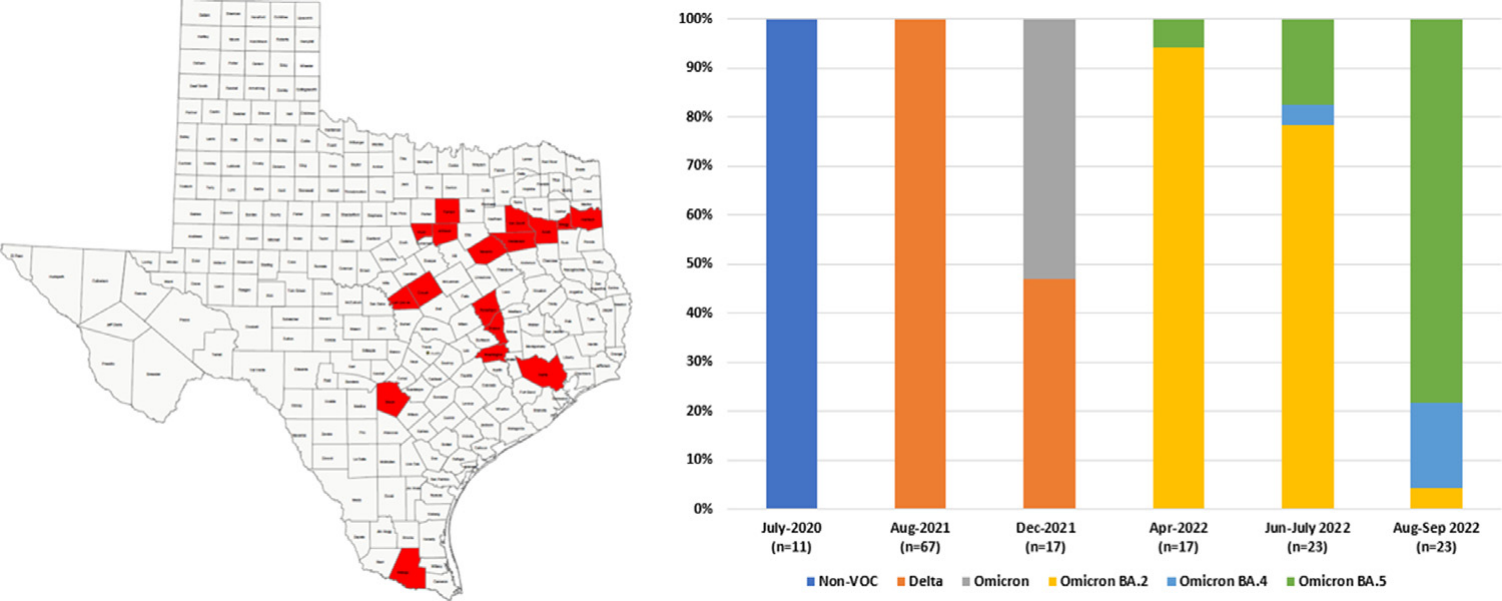
Evolution of SARS-CoV-2 variants in East Texas from July 2020-September 2022. *Reused from published article*[1]*and associated raw data is available as supplementary table-[1, sheet-2]*.

The data identified greater genomic diversity early in the pandemic, before classification of VOC. Initial samples from July 2020 identified the SARS-CoV-2 variants B.1; B.1.126; B.1.2; B.1.234; B.1.243; B.1.564; B.1.574; and B.1.602. The data revealed a progressive evolution from non-VOC infectivity with samples tested from July-Aug 2021 resulting in 100% calling for the WHO classified Delta VOC. Continued virus mutation confirmed co-circulation variants in samples tested from December 2021 with data revealing infectivity of Omicron (58%) and Delta (42%) variants. Whereas the data from April-September 2022 samples indicated Omicron responsible for 100% of infectivity with a dominant variant with an evolved dominance progression from Omicron BA.2 to Omicron BA.5 (Fig. 2).

The GISAID database for global SARS-CoV-2 sequence analysis, available on the Nexstrain server, was used to retrieve representative variant sequences [5]. All individual consensus genome sequence files were aligned using the Clustal-W Multiple Sequence Alignment Tool [6]. Phylogenetic analysis was performed using the Clustal Omega Server, and the phylogenetic tree was constructed using the Mega X tool with default maximum likelihood parameters [7,8].

## 3 Experimental Design, Materials and Methods

A total of 161 nasopharyngeal swab samples were collected from patients with positive SARS-CoV-2 qRT-PCR assay at Advanta Genetics in Tyler, Texas, USA (https://aalabs.com/). Total nucleic acid (NA) was extracted using the Roche MagNA Pure 96 system and Viral RNA Small Volume Kits (Port Scientific Inc, Canada). Isolated NA was archived at -80°C until library preparation. Whole genome synthetic RNAs from three reference strains (Omicron, Delta and Wuhan) were obtained from BEI Resources, and sequenced with each sequencing batch for quality control.

Libraries were prepared using the Illumina COVIDSeq protocol (Illumina Inc, USA). Briefly, first-strand cDNA was synthesized using reverse transcriptase and random hexamers primer. The SARS-CoV-2 genome was amplified using two sets of primers (COVIDSeq Primer Pool-1 and 2) in two multiplex PCR protocols. Libraries were constructed by tagmentation, and adapter ligation using IDT (Integrated DNA Technologies) for Illumina Nextera UD Index Set A. Individual libraries were quantified using Qubit 2.0 fluorometer (Invitrogen, Inc.) and pooled in equimolar concentration. Normalized library pools were sequenced. Final library pools were diluted to a 2 pM loading concentration, and dual-indexed paired-end sequencing of 75 bp reads was performed using an HO flow cell (150 cycles) on an Illumina MiniSeq® instrument.

Illumina baseSpace (https://basespace.illumina.com) bioinformatics was used for data QC, FASTQ generation, genome assembly and SARS-CoV-2 variant detection. Briefly, raw FASTQ files were trimmed and quality checked (Q > 30) using Basespace's FASTQ-QC application. QC-passed FASTQ files were aligned to the SARS-CoV-2 reference genome (NCBI reference sequence NC_045512.2) using the Bio-IT processor (version: 0 × 04261818). Basespace's DRAGEN COVID Lineage (version: 3.5.4) was used to determine the SARS-CoV-2 variant and to generate a single consensus FASTA file. Finally, individual consensus FASTA files were also analyzed for lineage assignment using the online version of Phylogenetic Assignment of Named Global Epidemic Lineages (PANGOLIN) (https://pangolin.cog-uk.io). Only consensus variants identified by both applications were used for further analysis.

## Ethics Statements

This research used de-identified residual samples, and the study was exempted by Institutional Review Board (IRB).

## Credit Author Statement

**Rob E. Carpenter:** Conceptualization, Investigation, Writing & editing; **Vaibhav K Tamrakar:** Data curation, writing & editing; **Sadia Almas:** Investigation, Methodology, Project administration; **Aditya Sharma:** Data curation, Formal analysis; **Rahul Sharma:** Conceptualization, Supervision, and Writing.

## Declaration of Competing Interests

The authors declare that they have no known competing financial interests or personal relationships that could have appeared to influence the work reported in this paper.

## Data Availability

SARS-CoV-2 Next Generation Sequencing (NGS) data from clinical isolates from the East Texas Region of the United States (Original data) (GISAID).

## References

[1] Carpenter R.E., Tamrakar V.K., Almas S., Brown E., Sharma R. (2022). COVIDSeq as laboratory developed test (LDT) for diagnosis of SARS-CoV-2 variants of concern (VOC). Arch. Clin. Biomed. Res..

[2] Carpenter R.E., Tamrakar V., Chahar H., Vine T., Sharma R. (2022). Confirming multiplex RT-qPCR use in COVID-19 with next-generation sequencing: strategies for epidemiological advantage. Glob. Health Epidemiol. Genom..

[3] R.E Chen, Zhang X., Case J.B., Winkler E.S., Liu Y., VanBlargan L.A. (2021). Resistance of SARS-CoV-2 variants to neutralization by monoclonal and serum-derived polyclonal antibodies. Nat. Med..

[4] Shen X., Tang H., Pajon R., Smith G., Glenn G.M., Shi W. (2021). Neutralization of SARS-CoV-2 variants B.1.429 and B.1.351. N. Engl. J. Med..

[5] Hadfield J., Megill C., Bell S.M. (2018). Nextstrain: real-time tracking of pathogen evolution. Bioinformatics.

[6] Sievers F., Wilm A., Dineen D. (2011). Fast, scalable generation of high-quality protein multiple sequence alignments using Clustal Omega. Mol. Syst. Biol..

[7] Sievers F, Wilm A, Dineen D (2011). Fast, scalable generation of high-quality protein multiple sequence alignments using Clustal Omega. Mol. Syst. Biol..

[8] Beck K.L., Seabolt E., Agarwal A. (2021). Semi-supervised pipeline for autonomous annotation of SARS-CoV-2 genomes. Viruses.

